# CREB Targets Define the Gene Expression Signature of Malignancies Having Reduced Levels of the Tumor Suppressor Tristetraprolin

**DOI:** 10.1371/journal.pone.0115517

**Published:** 2014-12-26

**Authors:** Mohammad Fallahi, Antonio L. Amelio, John L. Cleveland, Robert J. Rounbehler

**Affiliations:** 1 Informatics Core, The Scripps Research Institute, Scripps Florida, Jupiter, FL, United States of America; 2 Lineberger Comprehensive Cancer Center, School of Dentistry, University of North Carolina at Chapel Hill, Chapel Hill, NC, United States of America; 3 Department of Tumor Biology, H. Lee Moffitt Cancer Center and Research Institute, Tampa, FL, United States of America; Colorado State University, United States of America

## Abstract

The RNA-binding protein Tristetraprolin (TTP, ZFP36) functions as a tumor suppressor that impairs the development and disables the maintenance of MYC-driven lymphoma. In addition, other human cancers expressed reduced levels of *TTP*, suggesting that it may function as a tumor suppressor in several malignancies. To identify genes that may be associated with TTP tumor suppressor functions in human cancer, we analyzed The Cancer Genome Atlas (TCGA) breast cancer, lung adenocarcinoma, lung squamous cell carcinoma, and colon adenocarcinoma datasets. These analyses defined a signature of 50 genes differentially regulated between high and low *TTP*-expressing tumors. Notably, patients with low *TTP-*expressing breast cancer and lung adenocarcinoma had decreased survival rates and more aggressive tumors with increased necrosis. In addition, analysis across non-TCGA tumor gene expression databases identified a broad spectrum of human cancers having similarities with the *TTP*-low tumor gene signature, including pancreatic, bladder, and prostate cancer. TTP has documented roles in regulating mRNAs encoding inflammatory proteins, and pathway analysis identified several inflammatory pathways that are altered in tumors with low *TTP* expression. Surprisingly, the *TTP*-low tumor gene signature includes a core component of 20 under-expressed CREB target genes, suggesting that the regulation of CREB activity may be related to the tumor suppressor function of TTP. Thus, reduced levels of TTP are a potential biomarker for human cancers with poor outcome, and targeting the CREB pathway may be a therapeutic route for treating aggressive *TTP*-low tumors.

## Introduction

The Cancer Genome Atlas (TCGA) is a comprehensive project aimed at capturing the genomic and clinical details of more than 20 different types of cancer, with data from hundreds of patients for each tumor type [1]. Even though specific anatomical and histological features have historically defined cancer, in this age of genomics it has become evident that genetic alterations in tumors even within a given subtype are unique from one patient to another, underscoring the need for personalized cancer therapies. To move towards the ultimate goal of personalizing cancer treatment it is necessary to identify novel molecular biomarkers and therapeutic targets that serve as the basis for developing treatment options. Accordingly, here we utilized several TCGA datasets to define the gene expression signature of malignancies that express reduced levels of the newly discovered tumor suppressor Tristetraprolin (TTP, ZFP36) [2].

TTP functions in the post-transcriptional control of short-lived mRNAs having adenosine-uridine (AU)-rich elements (AREs) located in their 3′ untranslated regions (3′UTRs). Notably, genomic analysis has shown that at least 11% of all human genes contain AREs, and, pertinent to this study, all ten of the molecular mechanisms defined as the “Hallmarks of Cancer” include genes that contain AREs [3], [4]. AREs are bound by a class of proteins, including TTP, called AU-binding proteins (AUBPs), which either stabilize these transcripts or direct their destruction [5]. The NMR structure of TIS11D showed that the tandem zinc-finger domains, common to all TTP family members, bind to the nonameric 5′-UUAUUUAUU-3′ motif present in its mRNA targets; however, recent global analysis has shown that TTP can also bind to shorter ARE regions [6]–[8]. Upon binding, TTP transports its cargo to regions of the cell known as processing bodies (P-bodies), where the mRNA undergoes deadenylation, decapping, and degradation by a series of mRNA decay enzymes [9]. The role of TTP in post-transcriptional control was discovered from its ability to directly bind to an ARE present in the mRNA encoding the inflammatory cytokine tumor necrosis factor-α (TNFα), thereby promoting the decay of *TNFα* transcripts [10]. Indeed, *TTP* loss in knockout mice leads to supraphysiological levels of TNFα that, in turn, causes a severe autoimmune disease that manifests as erosive arthritis, dermatitis, cachexia and myeloid hyperplasia [11]. However, the ability of TTP to bind to *TNFα* mRNA can be suppressed by the p38/MK2 pathway, which phosphorylates and transiently inactivates TTP and causes it to translocate to the cytoplasm, allowing TNFα to be expressed [12], [13]. Further, several studies have shown important roles for TTP in regulating other inflammatory cytokines, and these have suggested that reduced levels of TTP may contribute to an array of human diseases where inflammation plays critical roles, including cancer [14].

A variety of tumor types and models have been used to assess the role of TTP in cancer. In a Myc-driven mouse model of B cell lymphoma, Myc represses *TTP* transcription. However, enforcing TTP expression in Myc-expressing B cells doubles the lifespan of these tumor-prone mice and disables maintenance of Myc-driven lymphoma; thus, in this scenario TTP functions as tumor suppressor [2]. Further, TTP loss in cervical cancer leads to stabilization of *E6-AP* ubiquitin ligase mRNA, and E6-AP triggers p53 degradation and the induction of hTERT, overriding senescence [15]. Also, reductions of TTP levels in colon cancer have been shown to lead to increased levels of the inflammatory cytokine *COX-2* and the pro-angiogenic cytokine *VEGF* [16], [17]. Moreover, low TTP levels correlate with high tumor grade and poor outcome in human breast cancer patients [18]. Finally, genomic analyses have revealed low levels of *TTP* in human glioma, head and neck squamous cell carcinoma, melanoma, and prostate cancer [18]–[21]. However, the targets of TTP that are required for its tumor suppressor functions remain undefined.

Here we applied genomic analyses using four TCGA tumor datasets (breast cancer, lung adenocarcinoma, lung squamous cell carcinoma, and colon adenocarcinoma) [22]–[24] to define the mRNA expression signature associated with reduced *TTP* levels in human malignancies. These analyses identified a shared signature, comprised of 50 genes, which are differentially expressed in high-*TTP* versus low-*TTP* expressing tumors. Notably, clinical data associated with these datasets establish that in some tumor types reduced *TTP* expression is a poor prognostic indicator that is associated with more aggressive and necrotic tumors. Unexpectedly, these analyses revealed that CREB target genes represent a significant proportion of the *TTP*-low tumor gene signature, suggesting that manipulating activity of the CREB pathway is a potential treatment option for patients with low *TTP* expressing tumors.

## Materials and Methods

### The Cancer Genome Atlas (TCGA) Data Retrieval

Breast cancer, lung adenocarcinoma, lung squamous cell carcinoma, and colon adenocarcinoma were downloaded from the TCGA portal (http://tcga-data.nci.nih.gov/). For expression profiling analyses, level 3 expression data of 20,475 genes and 73,599 isoforms from the RNASeqV2 platform were downloaded for each cancer dataset. For clinical analyses, a clinical matrix dataset was downloaded for each cancer. The number of samples included in each dataset at the time of these analyses was: breast cancer, 813; lung adenocarcinoma, 355; lung squamous cell carcinoma, 260; and colon adenocarcinoma, 193.

### Gene expression profiling analysis

RNA-Sequencing (RNA-Seq) by Expectation-Maximization (RSEM) normalized count was used to analyze gene-level or isoform level transcription estimates for the RNASeqV2 data from each TCGA dataset. For each cancer dataset, log2 normalized counts were imported into GeneSpring GX V12.1 (Agilent Technologies). Baseline transformation was set as the median for all samples. Upper and lower quartile groups (*TTP*-high and *TTP*-low) were defined based on *TTP* (*ZFP36*) expression within each dataset. Out of 20,475 genes in each RNA-Seq dataset, only genes that expressed higher than median in at least one sample were filtered for downstream analysis.

The GeneSpring Volcano Plot function was used to identify differentially expressed genes (DEGs) between the *TTP*-high and *TTP*-low groups for each TCGA dataset. Statistical test parameters were as follows: selected test, unpaired *t*-test; p-value computation, Asymptotic; multiple testing correction, Benjamini-Hichberg. Corrected p-value cut-off was set to 0.05 and fold change cut-off was set to 2.

The GeneSpring hierarchical clustering algorithm was used to generate heatmaps. The similarity measure was set to Pearson centered and the linkage rule was set to average. A Venn diagram was created using GeneSpring GX V12.1 software to evaluate which DEGs overlap between all four datasets to identify the *TTP*-low tumor gene signature.

Data from GSE32574 was used for gene expression analysis of unstimulated versus LPS-treated macrophages [25]. All genes shown on the heatmap were above the 50^th^ percentile in at least one sample.

### Analysis of clinical data

All clinical data analyzed herein are part of open access data generated from patient samples collected by TCGA. The clinical matrix dataset for each cancer type downloaded from the TCGA portal included overall survival or relapse-free survival, tumor subtype, tumor stage and tumor necrosis percentage. The TCGA breast cancer dataset included data for the presence and absence of estrogen receptor (ER), progesterone receptor (PR), and human epidermal growth factor receptor 2 (HER2), along with data for triple negative breast cancers (TNBCs). TCGA lung adenocarcinoma dataset included mutation data for *EGFR*, *ERBB4*, *KRAS* and *STK11*. Clinical data was analyzed for differences between tumors identified as having high *TTP* expression versus low *TTP* expression. Student's *t*-test was used to test for significance (p-value <0.05) of the tumor necrosis percentage analysis. Overall survival or recurrence-free survival data of patients was imported into GraphPad Prism V5.0 software (GraphPad Software, Inc.). The Mantel-Cox log-rank test (p-value <0.05) was used to test for significance.

### Identification of tumor sets having similarities to the *TTP*-low tumor gene signature

The NextBio Research platform (www.nextbio.com; Illumina, Inc.) was used to search thousands of human cancer-related mRNA biosets for significant overlap with the *TTP*-low tumor gene signature. Tumor sets with at least 10 samples and 25 genes shared with the *TTP*-low signature list were included in the list of significantly similar tumor datasets (p-value <0.05). Fisher's exact test was used to calculate the p-values.

### Identification of canonical pathways and upstream regulators of the *TTP*-low tumor gene signature

Ingenuity Pathway Analysis (IPA) software (Qiagen) was used to identify canonical pathways having significant overlap and upstream transcriptional regulators with significantly enriched targets of the *TTP*-low tumor gene signature. Fisher's exact test was used for assessing significance (p–value <0.05).

## Results

### Identification of a *TTP*-low tumor gene signature using The Cancer Genome Atlas

**Figure 1:**
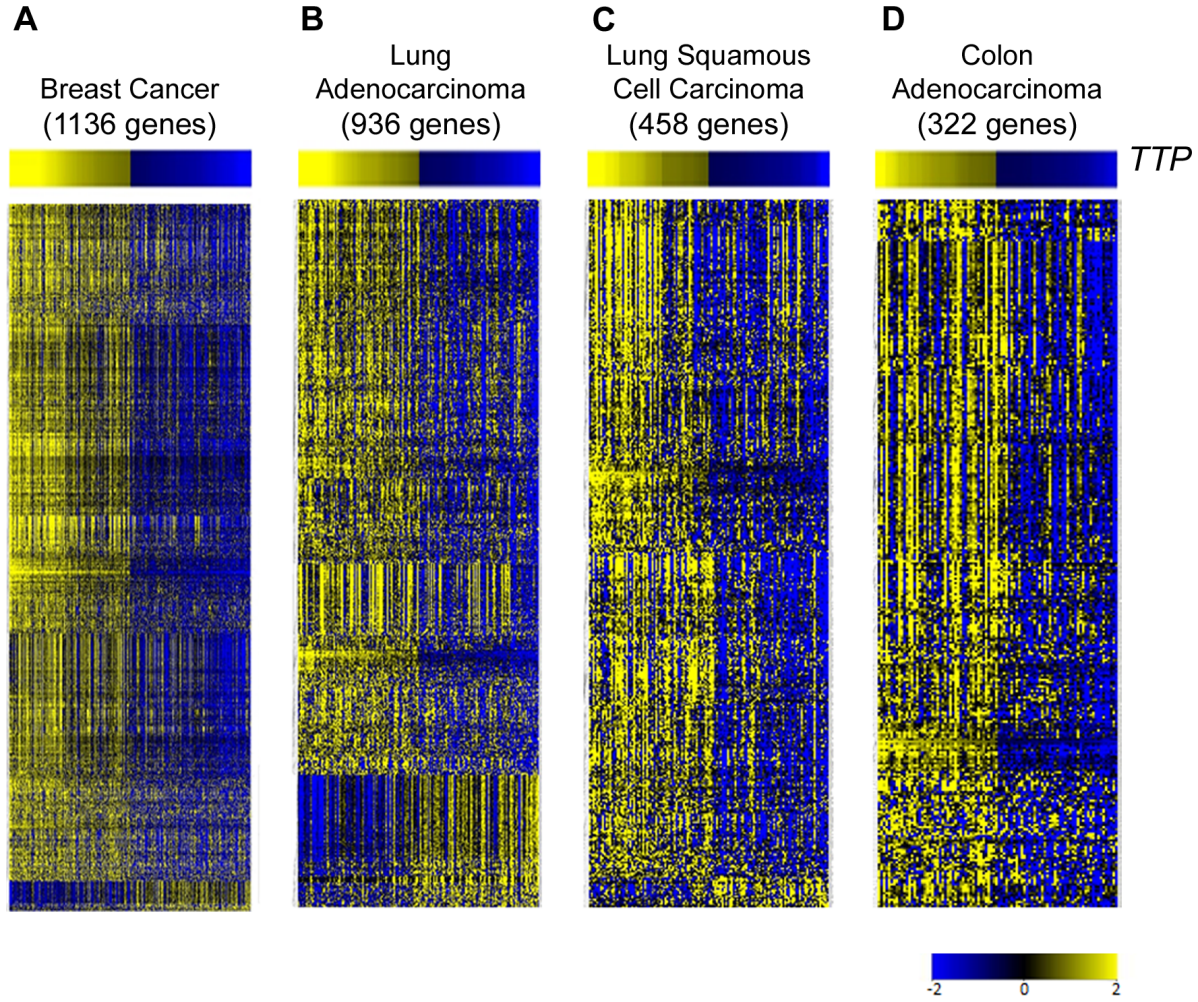
Differentially expressed genes between tumors in TCGA datasets with high and low expression of *TTP*. Gene expression profiling showing differentially expressed genes between *TTP*-high and *TTP*-low expressing tumors in TCGA breast cancer (**A**), lung adenocarcinoma (**B**), lung squamous cell carcinoma (**C**), and colon adenocarcinoma (**D**) databases. Information regarding the tumor samples in the *TTP*-high and *TTP*-low cohorts and the differentially expressed genes for each tumor type is presented in S1, S2, S3, S4, S5, S6, S7, S8 Tables. All genes shown are hierarchically clustered, have >2.0-fold change, and are significantly altered by unpaired *t*-test analysis (corrected p-value <0.05).

RNA Sequencing (RNA-Seq) data from TCGA breast cancer, lung adenocarcinoma, lung squamous cell carcinoma, and colon adenocarcinoma datasets [22]-[24] was analyzed, and each tumor type was divided into quartiles based on *TTP* expression levels (S1, S2, S3, S4 Tables). The *TTP*-high (top quartile) and *TTP*-low (bottom quartile) groups for each tumor were then analyzed for differentially expressed genes. These analyses revealed that the expression of hundreds of genes is altered in each tumor type between *TTP*-high and *TTP*-low tumors (Fig. 1; S5, S6, S7, S8 Tables).

**Figure 2:**
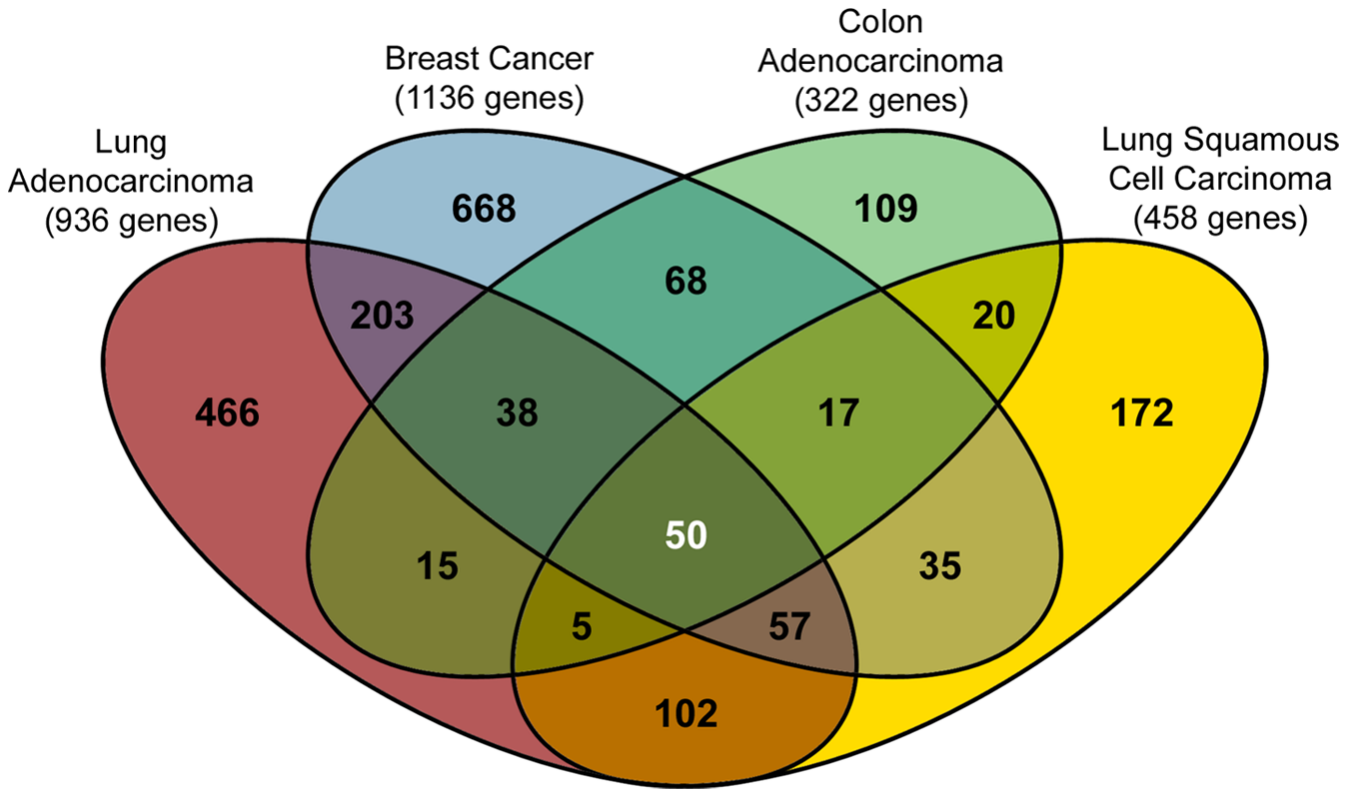
Identification of the *TTP*-low tumor gene signature. Venn diagram showing the overlap of differentially expressed genes between *TTP*-high and *TTP*-low expressing tumors. The center of the diagram indicates that there are 50 genes shared by all four cancer datasets that make up the *TTP*-low tumor gene signature. These 50 genes are listed in Table 1, and their fold change in each tumor dataset is listed in Table 2.

To identify genes whose expression is changed in all four tumor datasets, the sets of differentially expressed genes were compared, and a set of 50 genes was identified – the *TTP-*low tumor gene signature (Fig. 2; Table 1). As predicted, a large fraction (80%, 40 out of 50 genes) of the mRNAs in the *TTP*-low tumor gene signature have AREs in their 3′UTRs, including UUAUUUAUU nonamers, UAUUUAUU octamers and AUUUA pentamers that have been shown to be bound by TTP in global analyses (Table 1) [8]. Interestingly, though the role of TTP as an mRNA destabilizing AUBP predicted that TTP-target genes would be increased in the *TTP*-low cohort, the expression of all 50 of these signature genes is markedly reduced in *TTP*-low tumors compared to *TTP*-high tumors in the TCGA breast cancer, lung adenocarcinoma, lung squamous cell carcinoma and colon adenocarcinoma datasets (Table 2). Thus, other regulatory factors in these malignancies may be involved in controlling the expression of genes of the *TTP-*low tumor gene signature, and it is possible that such factors are common to all *TTP*-low tumors (see below).

**Table 1:** AREs located in genes in the *TTP*-low tumor gene signature.

Gene Symbol	ARE in 3′UTR^a^	ARE Nonamers (UUAUUUAUU)^a^	ARE Octamers (UAUUUAUU)^a^	ARE Heptamers (UAUUUAU)^a^	ARE Pentamers (AUUUA)^a^
*ADAMTS1*	Yes	0	1	0	3
*ADAMTS8*	Yes	0	0	0	1
*ADH1B*	Yes	0	0	0	7
*AG2*	Yes	0	0	0	3
*C16orf89*	No	0	0	0	0
*C3*	No	0	0	0	0
*C8orf4*	Yes	1	0	0	4
*CADM3*	No	0	0	0	0
*CH25H*	Yes	0	1	0	2
*CSF3*	Yes	0	0	0	7
*CSRNP1*	Yes	1	0	0	3
*CTGF*	Yes	0	0	0	3
*CXCL2*	Yes	5	2	0	1
*CYR61*	Yes	0	0	0	5
*DUSP1*	Yes	2	0	0	1
*EDN1*	Yes	0	0	1	3
*EGR1*	Yes	0	0	0	1
*EGR2*	Yes	0	0	0	2
*EGR3*	Yes	0	0	0	3
*FABP4*	Yes	0	0	0	2
*FOS*	Yes	1	0	1	3
*FOSB*	Yes	0	0	0	3
*GADD45B*	No	0	0	0	0
*HBA1*	No	0	0	0	0
*HBA2*	No	0	0	0	0
*HBB*	Yes	0	0	0	1
*HBEGF*	Yes	0	1	1	3
*HP*	No	0	0	0	0
*IL6*	Yes	0	0	2	4
*JUN*	Yes	0	0	1	2
*JUNB*	Yes	0	0	0	2
*KLF2*	Yes	0	0	0	3
*KLF6*	Yes	0	0	0	6
*NR4A1*	Yes	0	0	0	3
*NR4A2*	Yes	0	0	0	4
*NR4A3*	Yes	0	0	1	9
*OSM*	Yes	0	0	1	4
*PTGDS*	No	0	0	0	0
*PTGS2*	Yes	1	1	3	17
*RRAD*	No	0	0	0	0
*SCARA5*	Yes	0	0	0	2
*SELE*	Yes	0	1	1	6
*SERPINE1*	Yes	0	0	0	4
*SIK1*	Yes	0	0	0	3
*SLC6A14*	Yes	1	0	0	7
*SLIT3*	Yes	0	0	0	1
*SOCS3*	Yes	0	0	2	2
*TPSB2*	No	0	0	0	0
*TTP (ZFP36)*	Yes	1	0	0	4
*VSIG2*	Yes	0	0	0	1

a Annontated by AREsite 1.0 (http://rna.tbi.univie.ac.at/cgi-bin/AREsite.cgi) [46].

**Table 2:** Fold change in gene expression between high and low *TTP-*expressing tumors in TCGA datasets.

Gene Symbol	Fold Change in Breast Cancer	Fold Change in Lung Adenocarcinoma	Fold Change in Lung Squamous Cell Carcinoma	Fold Change in Colon Adenocarcinoma
*ADAMTS1*	3.18	2.79	2.17	2.48
*ADAMTS8*	2.53	5.19	2.36	2.03
*ADH1B*	19.24	9.02	2.96	4.13
*AG2*	3.22	3.77	3.17	2.63
*C16orf89*	2.19	3.81	3.21	2.33
*C3*	2.80	3.09	2.13	2.28
*C8orf4*	3.26	3.46	2.41	2.64
*CADM3*	5.81	2.25	2.07	3.39
*CH25H*	4.62	2.86	2.75	2.25
*CSF3*	2.16	4.02	6.38	2.84
*CSRNP1*	2.68	3.20	2.41	2.55
*CTGF*	3.31	2.38	2.27	2.19
*CXCL2*	6.61	5.22	4.79	2.41
*CYR61*	5.23	2.27	2.99	3.53
*DUSP1*	7.28	6.84	4.19	5.12
*EDN1*	3.70	2.73	2.46	2.34
*EGR1*	9.44	4.61	4.17	7.23
*EGR2*	5.38	2.26	2.11	3.79
*EGR3*	5.66	3.83	3.21	5.38
*FABP4*	10.53	3.28	2.86	3.40
*FOS*	13.74	7.00	5.85	8.08
*FOSB*	22.65	22.91	7.32	12.64
*GADD45B*	2.33	2.92	2.45	2.11
*HBA1*	3.84	6.26	5.01	4.25
*HBA2*	3.25	4.72	2.65	2.46
*HBB*	4.23	4.48	2.34	3.30
*HBEGF*	2.79	2.99	2.30	3.58
*HP*	3.11	3.21	3.73	2.91
*IL6*	9.52	2.81	3.14	5.80
*JUN*	3.68	2.11	2.44	2.04
*JUNB*	3.53	2.51	2.84	3.41
*KLF2*	3.23	3.36	2.13	3.00
*KLF6*	2.19	2.06	2.06	2.31
*NR4A1*	5.54	7.11	3.77	4.41
*NR4A2*	2.42	5.52	2.77	2.74
*NR4A3*	5.00	5.27	3.63	3.77
*OSM*	2.90	2.42	2.32	3.93
*PTGDS*	3.80	2.60	2.14	2.19
*PTGS2*	5.36	2.15	2.07	3.26
*RRAD*	3.56	2.77	2.97	2.92
*SCARA5*	7.60	3.80	2.44	2.60
*SELE*	4.12	2.14	3.03	2.37
*SERPINE1*	3.72	2.09	2.57	2.95
*SIK1*	2.48	3.30	2.33	2.44
*SLC6A14*	2.47	3.88	3.29	2.86
*SLIT3*	2.24	3.00	2.45	2.32
*SOCS3*	3.41	2.63	2.38	3.27
*TPSB2*	3.00	2.54	2.26	2.28
*VSIG2*	3.24	4.19	2.48	4.48
*TTP (ZFP36)*	8.07	7.32	5.69	4.98

### Low *TTP* expression is a poor prognostic indicator in breast cancer and lung adenocarcinoma

**Figure 3:**
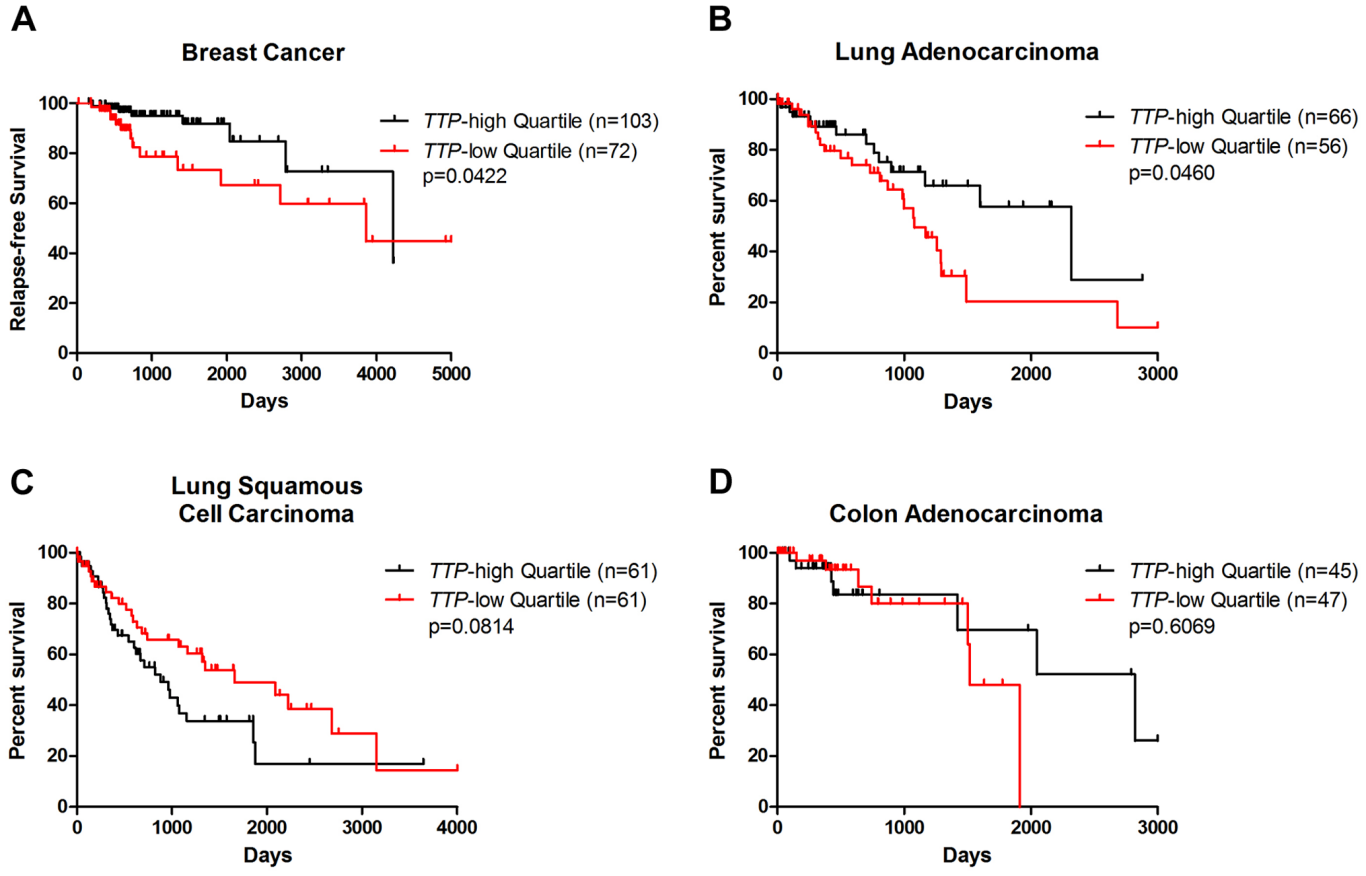
Low expression of *TTP* connotes poor outcome for breast cancer and lung adenocarcinoma patients. Relapse-free survival data from TCGA breast cancer (**A**) and overall survival data from TCGA lung adenocarcinoma (**B**), lung squamous cell carcinoma (**C**), and colon adenocarcinoma (**D**) was used to generate Kaplan-Meier survival curves of *TTP*-high versus *TTP*-low patients in each dataset. The p-values were determined by the Mantel-Cox log-rank test.

To determine the association of low *TTP* expression with patient outcome in these cancers, available TCGA survival data were analyzed between the *TTP*-high and *TTP-*low tumor sets. For the TCGA breast cancer clinical dataset, only a few overall survival events are currently available [22]. However, relapse-free survival data is available, and analysis of this data shows that breast cancer patients with low levels of *TTP* expression have a higher incidence of relapse than their *TTP*-high counterparts (Fig. 3A). This confirms previous findings suggesting that low *TTP* expression is a poor prognostic indicator in breast cancer [18].

TCGA data was also used to compare overall survival rates between *TTP*-high and *TTP-*low patients for the three other cancer types. For lung adenocarcinoma, patients with low levels of *TTP* expression have decreased survival rates compared to *TTP*-high cohorts (Fig. 3B). However, there were no significant differences in survival rates of *TTP-*low versus *TTP*-high cohorts in lung squamous cell carcinoma or colon adenocarcinoma patients (Figs. 3C and D). Thus, in some (*e.g.*, breast cancer and lung adenocarcinoma), but not all, cancer types patients with tumors having decreased levels of *TTP* have a worse outcome.

Genomic analysis of human cancer datasets using NextBio Research software identified another 260 datasets, which compared either cancer tissue to normal tissue, cancer adjacent tissue to normal tissue, or differential tumor populations, as having significant similarities to the *TTP*-low tumor gene signature (S9 Table). In addition to breast, lung, and colon cancers, other malignancies having similarities to the *TTP*-low tumor gene signature include uterine, pancreatic, liver, bladder and prostate cancers (Table 3). Thus, TTP might be an important diagnostic biomarker for predicting patient outcome in a broad spectrum of human tumor types.

**Table 3:** Top 20 other human cancers with similarities to the *TTP*-low tumor gene signature.

Bioset	Number of genes shared^a^	p-Value
Normal gastric tissue tumor adjacent _vs_ normal gastric tissue from healthy individuals	27	1.80E-42
Uterine leiomyomata neoplasm fibroid without 7q deletion _vs_ normal myometrium	37	1.30E-37
Uterine leiomyoma samples _vs_ adjacent normal myometrium	37	4.60E-37
Uterine leiomyomata neoplasm fibroid with 7q deletion _vs_ normal myometrium	31	4.60E-35
Human uterine leiomyomata fibroid _vs_ adjacent normal myometrium_GPL1355	37	8.40E-35
Pancreatic cancer sample _vs_ non-malignant adjacent pancreatic tissue	32	1.20E-33
Uterine cervix - low grade squamous intraepithelial lesions _vs_ normal tissue_GPL571	27	5.70E-32
Adult germ cell carcinoma - Teratoma _vs_ normal testis	46	1.50E-30
Differentiated hepatocellular carcinoma Grade I-II _vs_ normal liver	29	2.30E-30
Soft-tissue samples of all sarcoma patients _vs_ healthy adipose controls	37	1.90E-26
Bladder urothelial cell carcinoma samples _vs_ non-cancerous samples	38	2.30E-26
Human adrenocortical adenoma _vs_ normal adrenal cortex	33	1.30E-25
Adrenal cortex - adrenocortical adenoma _vs_ normal tissue	33	1.30E-25
Prostate Cancer Pathological Gleason Score 8-10 _vs_ Gleason Score 5-6	28	3.00E-25
Soft-tissue samples of malignant fibrous histiocytoma patients _vs_ healthy adipose controls	38	2.10E-24
Soft-tissue samples of MFH-myxofibrosarcoma patients _vs_ healthy adipose controls	35	3.30E-24
Human adrenocortical carcinoma _vs_ normal adrenal cortex	36	1.90E-23
Adrenal cortex - adrenocortical carcinoma _vs_ normal tissue	36	1.90E-23
Soft-tissue samples of leiomyosarcoma patients _vs_ healthy adipose controls	39	3.10E-23
Thyroid tumors _vs_ adjacent matched normal thyroid biopsies	29	3.30E-23

a Minimum 25 genes shared between the bioset and the *TTP*-low tumor gene signature.

### Biomarkers in *TTP*-low breast cancer and lung adenocarcinoma

**Figure 4:**
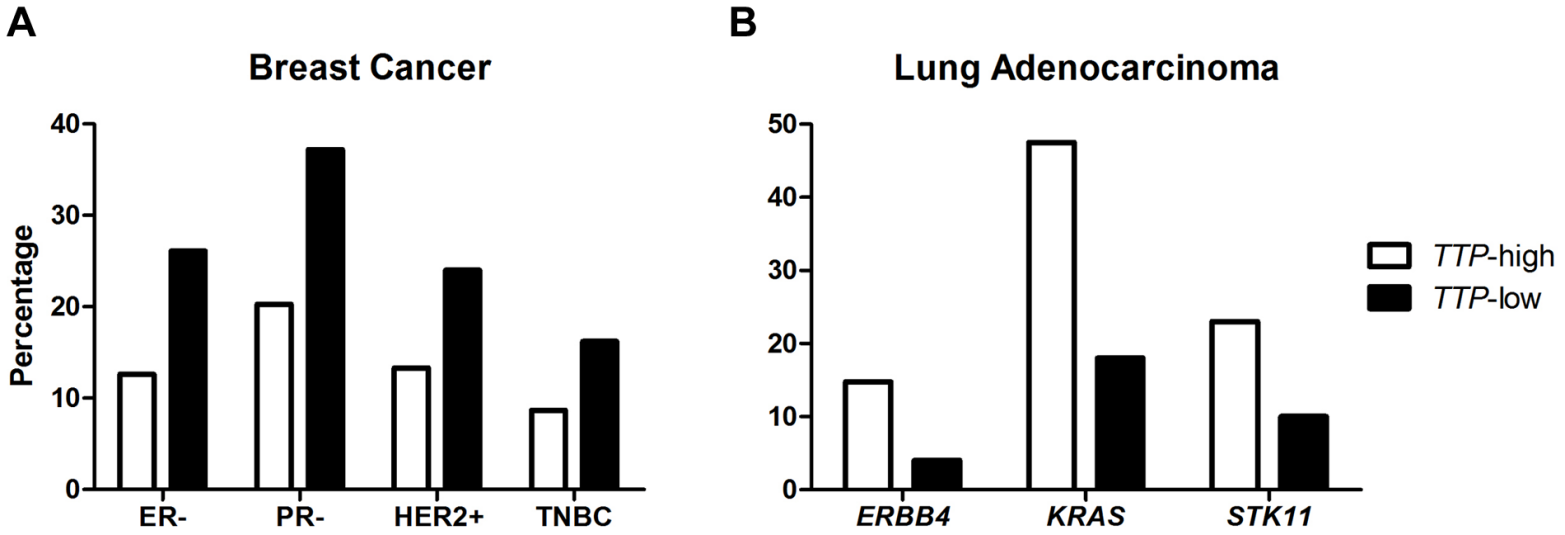
Differences in breast cancer biomarkers and lung adenocarcinoma mutations based on *TTP* expression levels. (**A**) Percentages of estrogen receptor-negative (ER-), progesterone receptor-negative (PR-), human epidermal growth factor receptor 2-enriched-positive (HER2+), and triple negative breast cancer (TNBC) patients in the *TTP*-high and *TTP*-low TCGA breast cancer sets. (**B**) Percentages of *ERBB4*, *KRAS*, and *STK11* mutations in *TTP*-high and *TTP*-low TCGA lung adenocarcinoma patients.

Specific proteins and/or genes are clinically proven diagnostic markers for classifying tumor subtypes, predicting patient outcomes, and developing treatment plans. For example, breast tumors are commonly assessed for three receptors that are used as pathological biomarkers: the presence or absence of estrogen receptor (ER) and progesterone receptor (PR), and the enrichment of human epidermal growth factor receptor 2 (HER2 or ERBB2) [26], [27]. In the TCGA breast cancer dataset, *TTP*-low tumors are twice as likely to be ER-negative (ER-) or PR-negative (PR-) versus *TTP*-high tumors, indicating that hormone therapies commonly used to block estrogen activity might be less effective at impairing the growth of breast tumors with low *TTP* expression (Fig. 4A). Conversely, twice as many *TTP*-low breast cancers are HER2-positive (HER2+) compared to the *TTP*-high cohort, indicating that such patients might have an improved response to trastuzumab (Herceptin), which targets HER2 [28]. Further, *TTP*-low breast cancers have approximately two times more triple-negative breast cancers (TNBCs) than *TTP*-high tumors. TNBCs lack the presence of all three receptors, and have limited treatment options and poor overall outcome [29].

Expression profiling has identified three molecular subtypes of lung adenocarcinoma, bronchioid, magnoid, and squamoid [30]. *EGFR* mutations are more frequent in bronchioid tumors, whereas *TP53*, *KRAS*, and *STK11* (the gene encoding the tumor suppressor LKB1) mutations are more frequent in magnoid tumors [31]. TCGA clinical data was analyzed for mutations of these genes in *TTP*-high versus *TTP*-low lung adenocarcinomas (except for *TP53*, which was not included in this dataset [24]). No difference was found for the mutation frequency of *EGFR* between the two *TTP* groups (data not shown). However, the EGFR family member *ERBB4* is less commonly mutated in *TTP-*low than *TTP*-high tumors (Fig. 4B). Similarly, *KRAS* and *STK11* mutations are less frequent in low *TTP-*expressing lung adenocarcinomas than in the high *TTP*-expressing cohort. Thus, even though the prognosis of patients having *TTP*-low expressing lung adenocarcinomas is worse (Fig. 3B), the classifying mutations of this malignancy are much less frequent in this cohort, suggesting that other alterations, or perhaps reductions in TTP alone, are drivers of these tumors.

### Low levels of *TTP* correlate with more aggressive subtypes of breast cancer, lung adenocarcinoma and lung squamous cell carcinoma

**Figure 5:**
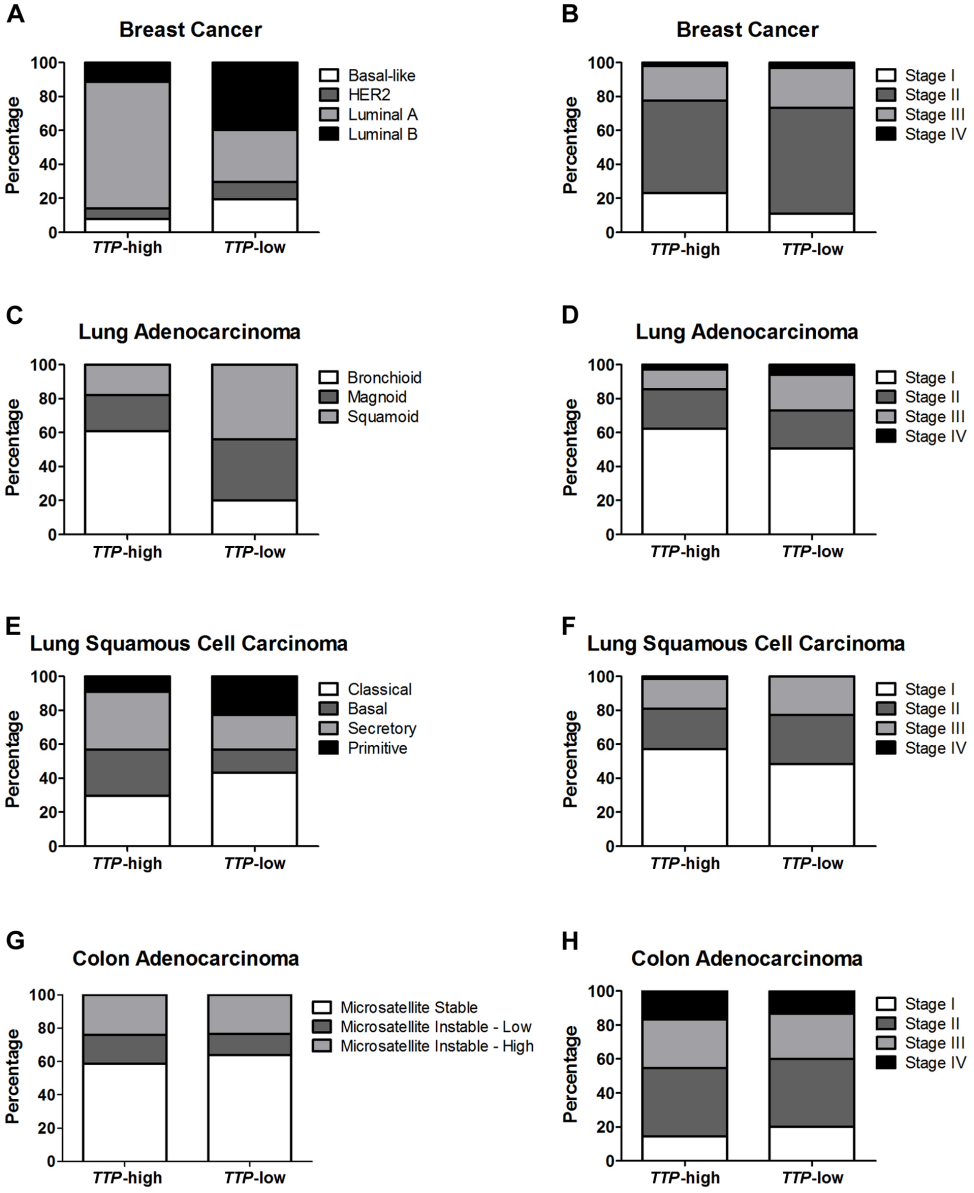
Low *TTP* expression correlates with more aggressive tumor subtypes and advanced tumor stage. Percentages of tumor expression subtypes (*left column*) and tumor stage (*right column*) are shown for patients in the *TTP*-high and *TTP*-low groups for TCGA breast cancer (**A and B**), lung adenocarcinoma (**C and D**), lung squamous cell carcinoma (**E and F**), and colon adenocarcinoma (**G and H**) datasets.

Breast cancer is divided into four molecular subtypes, luminal A, luminal B, HER2, and basal-like, based on the expression of ER, PR, and HER2 [26], [27]. Luminal A and luminal B are ER-positive (ER+) subtypes, and, in general, luminal B breast tumors are more aggressive and these patients have a worse prognosis versus patients with luminal A tumors [27]. The HER2 and basal-like subtypes are both ER-negative forms of breast cancer [26]. Further most, but not all, basal-like breast cancers are also TNBCs [32]. The TCGA breast cancer database includes tumors from all four subtypes, and the percentage of each in the *TTP-*high and *TTP*-low quartiles was calculated (Fig. 5A). Comparing these subtypes between the two groups, only luminal A, the subtype with the best prognosis, has a higher percentage in *TTP-*high (71%) than *TTP-*low tumors (30%). In contrast, in *TTP*-low breast tumors, the percentage of luminal B (40%) and basal-like (19%) subtypes is much higher than in the *TTP-*high cohort (11% and 7%, respectively). Finally, patients in the *TTP-*low group present more with Stage II (62%) and less with Stage I tumors (11%) compared to the *TTP-*high patients (55% and 23%, respectively) (Fig. 5B). Thus, low expression of *TTP* in breast cancer correlates with more aggressive tumor types.

Bronchioid lung adenocarcinoma has the most favorable patient outcome of the three lung adenocarcinoma subtypes [30], [31]. Notably, over 60% of the patients having high levels of *TTP* expressed in their tumors were of the bronchioid subtype, while only 20% of *TTP-*low patients have this favorable subtype (Fig. 5C). In contrast, magnoid and squamoid tumors, which have similar overall survival rates, are much more prevalent in *TTP-*low lung adenocarcinoma patients (36% and 44%, respectively) than their *TTP-*high counterparts (21% and 18%). This is particularly noteworthy for squamoid lung adenocarcinoma, as analyses of this subtype have yet to identify any characteristic genomic alterations [31]. Finally, nearly twice as many *TTP-*low lung adenocarcinoma patients have Stage III or Stage IV tumors (27% combined) than *TTP*-high patients (14% combined) (Fig. 5D). Therefore, like in breast cancer, lung adenocarcinomas with low expression of the tumor suppressor gene *TTP* are much more likely to have more aggressive and advanced tumors.

There are four expression subtypes of lung squamous cell carcinoma, primitive, classical, secretory, and basal [33]. The classical, secretory, and basal subtypes have similar patient outcomes, and the classical form accounts for the largest percentage of the *TTP*-low group (43%) versus the *TTP*-high cohort (30%) (Fig. 5E). In contrast, secretory or basal lung squamous cell carcinomas were more likely to have high levels of *TTP* (27% and 34%, respectively) than tumors with low *TTP* (14% and 20%, respectively). Importantly, the primitive subtype of lung squamous cell carcinoma are poorly differentiated and have the worse prognosis [33], and patients with low *TTP* expression have this detrimental form more often (23%) than individuals with high *TTP* expression (9%). Finally, lung squamous cell carcinoma patients with low *TTP* levels have a higher percentage of Stage II, III and IV tumors (52% combined) than *TTP-*high patients (43% combined) (Fig. 5F). Thus, although there are no differences in overall survival between *TTP*-high and *TTP*-low lung squamous cell carcinoma patients, there are differences in clinical phenotypes that suggest that reduced *TTP* expression may contribute to the aggressiveness of these tumors.

Colon adenocarcinomas are comprised of two biological subtypes, the microsatellite instability group, which have high levels of hypermethylation, and the microsatellite stable group, which lack hypermethylation but are chromosomally unstable [23]. There was no difference in the occurrence of these phenotypes between the *TTP*-high and *TTP*-low cohorts in the TCGA colon adenocarcinoma dataset (Fig. 5G). Further, the stages of colon tumors having high and low *TTP* expression were similar (Fig. 5H). Thus, *TTP* expression does not correlate with colon adenocarcinoma subtypes.

### *TTP*-low tumor signature genes affect inflammatory pathways

To identify mechanistic pathways that might be altered in *TTP*-low tumors, Ingenuity Pathway Analysis (IPA) software was applied to the gene signature. This revealed that 16 of the top 20 pathways affected by *TTP* expression levels are inflammatory pathways (Table 4, S10 Table) in accord with data showing that decreased levels of TTP result in increased inflammation [11], [14]. Further, *TTP* expression is induced by ligands that trigger the innate immune response [10], and five of the inflammatory pathways affected by this gene signature are also innate immunity pathways. Lipopolysaccharide (LPS) is well-established as an inducer of innate immunity, and gene expression profiling of macrophages stimulated by LPS compared to unstimulated macrophages [25] found that several genes in the *TTP*-low tumor signature are altered during activation of the innate immune pathway in a fashion similar to cancer based on *TTP* expression (S1 Fig.). Finally, 14 of the genes in the *TTP*-low tumor gene signature are classified as innate immune genes by the InnateDB database (www.innatedb.com) [34]. This suggests that TTP is specifically involved in controlling inflammation that is directed by the innate immune response.

**Table 4:** Top 20 canonical pathways significantly altered by the *TTP*-low tumor gene signature.

Ingenuity Canonical Pathways	p-value	Inflammatory Pathway	Innate Immune Pathway
Acute Phase Response Signaling	2.63E-07	Yes	No
Glucocorticoid Receptor Signaling	4.79E-06	Yes	No
IGF-1 Signaling	5.37E-06	No	No
IL-10 Signaling	2.95E-05	Yes	No
Role of JAK family kinases in IL-6-type Cytokine Signaling	3.72E-05	Yes	No
HMGB1 Signaling	1.02E-04	Yes	No
IL-17A Signaling in Fibroblasts	1.05E-04	Yes	Yes
HGF Signaling	1.35E-04	No	No
Role of Macrophages, Fibroblasts and Endothelial Cells in Rheumatoid Arthritis	1.38E-04	Yes	No
MIF Regulation of Innate Immunity	1.70E-04	Yes	Yes
Role of Tissue Factor in Cancer	2.04E-04	Yes	No
Corticotropin Releasing Hormone Signaling	2.19E-04	Yes	No
IL-6 Signaling	2.40E-04	Yes	Yes
Prostanoid Biosynthesis	2.40E-04	Yes	No
Hepatic Fibrosis/Hepatic Stellate Cell Activation	4.79E-04	Yes	No
Erythropoietin Signaling	7.24E-04	No	No
JAK/Stat Signaling	7.24E-04	Yes	No
IL-17 Signaling	8.91E-04	Yes	Yes
Prolactin Signaling	9.33E-04	No	No
Differential Regulation of Cytokine Production in Macrophages and T Helper Cells by IL-17A and IL-17F	1.00E-03	Yes	Yes

**Figure 6:**
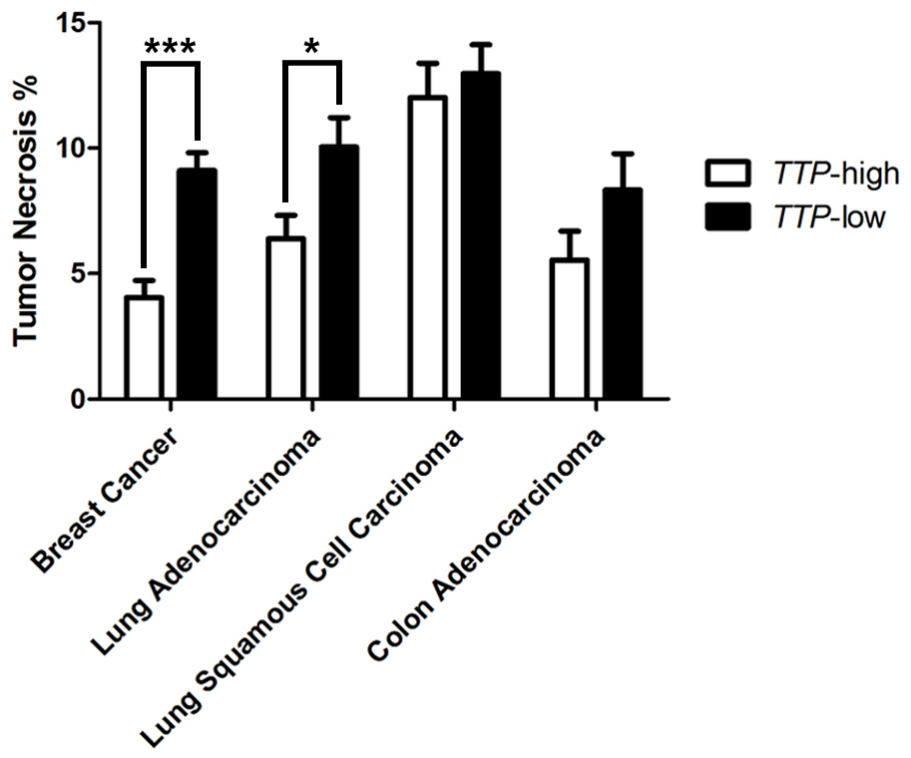
Low *TTP* levels correlate with increased tumor necrosis in breast cancer and lung adenocarcinoma. Average percentages of tumor necrosis in *TTP*-high and *TTP*-low tumors in TCGA breast cancer, lung adenocarcinoma, lung squamous cell carcinoma, and colon adenocarcinoma datasets. The p-values were determined by Student's *t*-test (*p<0.05, *** p<0.001).

The presence of necrosis within tumors is linked with aggressive disease and increased inflammation [35], [36]. Indeed, histological examination of tumor samples in the TCGA datasets revealed that *TTP*-low breast cancers and lung adenocarcinomas have significantly more tumor necrosis than *TTP*-high tumors (Fig. 6). In contrast, no differences in tumor necrosis were found in lung squamous cell carcinoma and colon adenocarcinoma. Collectively, these data suggest that TTP plays a critical role in regulating tumor inflammation in at least in some malignancies.

### CREB target genes are a core component of the *TTP*-low tumor gene signature

**Figure 7:**
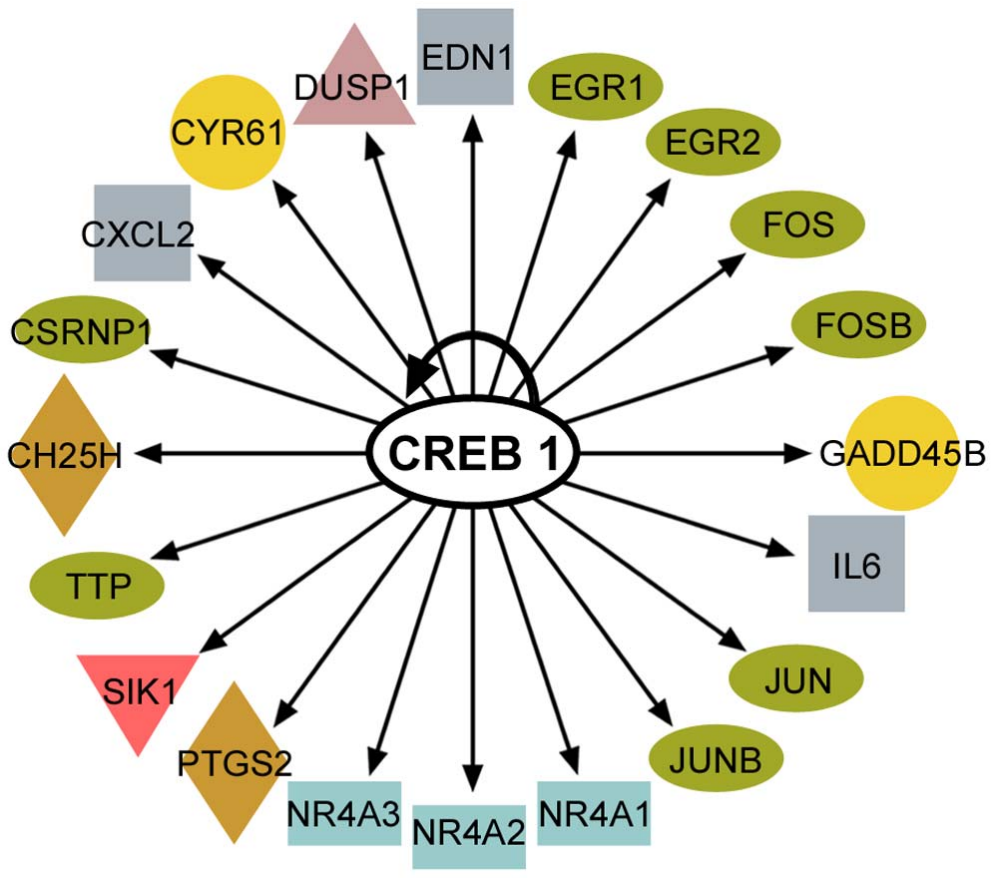
CREB-target genes are a core component of the *TTP*-low tumor gene signature. Diagram of the 20 CREB-target genes found by Ingenuity Pathway Analysis in the *TTP*-low tumor gene signature.

**Figure 8:**
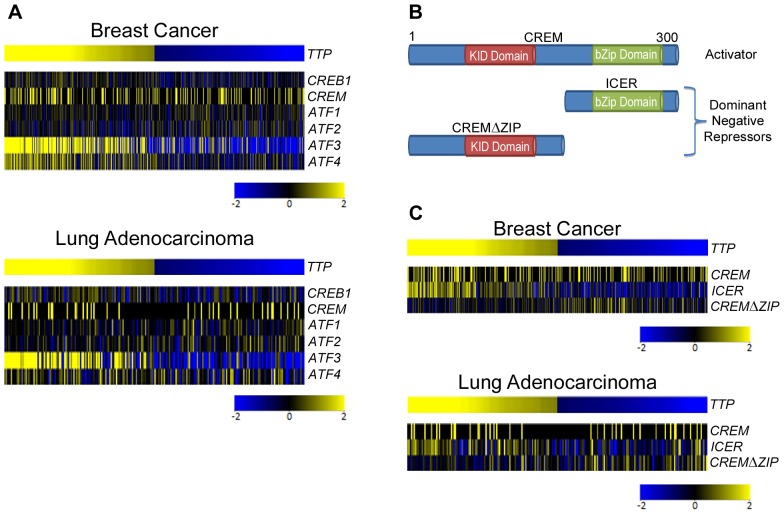
Expression of CREB family members in breast cancer and lung adenocarcinoma based on *TTP* levels. Gene expression profiling showing the expression levels of canonical *CREB* family members (**A**), and comparing the expression levels of *CREM* versus its dominant negative splice variants *ICER* and *CREMΔZIP* (**C**) in *TTP*-high and *TTP*-low expressing TCGA breast cancers and lung adenocarcinomas. (**B**) Cartoon showing the canonical CREM protein and its dominant negative splice variants ICER and CREMΔZIP.

IPA software was also used to identify transcription factors that control the expression of the signature genes in tumors with reduced *TTP* levels. Surprisingly, the cyclic AMP response element (CRE)-binding protein (CREB) family of activators (CREB1, CREM, ATF1) was revealed as the top upstream regulator, where it directly regulates the transcription of 20 out of the 50 genes in the signature (Table 5; S11 Table; Fig. 7). This suggests a previously unknown association between the activity of the CREB family of transcription factors and the ability of TTP to function as a tumor suppressor. All 20 of the CREB targets were repressed, despite the fact that *CREB* expression was not significantly altered in *TTP*-low versus *TTP*-high tumors (Fig. 8A; S2 Fig.). However, the expression level of the CREB family member *Activating Transcription Factor 3* (*ATF3*) was significantly reduced in *TTP*-low breast cancer, lung adenocarcinoma and colon adenocarcinoma. Interestingly, ATF3 expression is induced by innate signaling and functions to harness this response [25], [37]. In scenarios where there is chronic stimulation of innate immune signaling, such as that provoked by high levels of necrosis, one would predict innate immune tolerance [38] resulting in reductions of *ATF3* expression, which is precisely what is observed in the necrosis-associated *TTP*-low gene expression signature.

**Table 5:** Top 10 upstream regulators of the *TTP*-low tumor gene signature.

Upstream Regulator	Number of molecules in the *TTP*-low tumor gene signature^a^	p-value
CREB1	20	6.77E-27
CREBBP	17	3.89E-23
FOS	17	3.53E-16
SMAD3	12	1.14E-14
CREM	11	2.01E-14
NFκB (complex)	16	6.72E-14
ELK4	6	1.10E-13
PPARγ	14	1.30E-13
STAT3	14	1.72E-13
NR3C2	9	5.33E-13
FOSL1	8	7.49E-13
CEBPα	13	1.45E-12
ELK1	7	1.96E-12
JUN	13	2.18E-12
RELA	12	9.28E-12

a Minimum 3 genes in the *TTP*-low tumor gene signature must be targets of the upstream regulator.

Some splice variants of CREB family members function as dominant negative repressors of CREB activity [39], [40]. Thus, we assessed if the repression of CREB target genes in the *TTP*-low tumor gene signature was associated with alterations in the levels of the well-characterized cAMP Response Element Modulator (CREM) variant known as Inducible cAMP Early Repressor (ICER). ICER contains a basic leucine zipper (bZIP) domain that directs binding to CRE sites but lacks the *N*-terminal domain that binds to regulatory cofactors necessary for CREB transcriptional activity (Fig. 8B). *ICER* expression significantly correlated with *TTP* in all four cancer datasets; thus, it is unlikely to impair the transcriptional activity of CREB family members in *TTP*-low tumors (Fig. 8C; S3 Fig.). However, the expression of *CREM transcript variant 2*, which harbors the *N*-terminal domain of CREM but lacks the bZIP domain (CREMΔZIP) and is predicted to compete with CREB family members for necessary transcriptional cofactors (Fig. 8B), was significantly increased in breast cancer, lung adenocarcinoma, and lung squamous cell carcinoma having low *TTP* expression (Fig. 8C). Therefore, CREMΔZIP may impair the transcriptional activity of CREB family members in some *TTP*-low cancers and lead to the repression of CREB target genes in these tumors.

## Discussion

The identification of the *TTP*-low tumor gene signature in the TCGA breast cancer, lung adenocarcinoma, lung squamous cell carcinoma and colon adenocarcinoma datasets provides new avenues for investigating the functions of TTP as a tumor suppressor across a broad spectrum of human malignancies. In some scenarios, such as breast cancer and lung adenocarcinoma, reduced levels of *TTP* are associated with worse patient outcome, more aggressive tumor stage and subtypes, and increased tumor necrosis. In addition, the expression of genes in the *TTP-*low tumor gene signature is altered in a similar fashion in hundreds of other human tumor datasets, including uterine, pancreatic, bladder, liver, and prostate cancers. For most of these tumor types little is known regarding potential roles of TTP, and in the case of pancreatic and bladder cancer this is the first time any connection to TTP has been described. Therefore, TTP should be thoroughly explored across an array of human cancers, especially in more aggressive subtypes and/or in tumors having high levels of tumor necrosis, to determine if low *TTP* expression levels are a prognostic indicator.

The analyses herein show that the *TTP*-low tumor gene signature is involved in inflammatory pathways, particularly innate immunity, and the increased necrosis in tumors classified as *TTP*-low supports this notion. Necrotic cells release endogenous ligands called damage-associated molecular patterns (DAMPs), which bind to and activate Toll-like receptors (TLRs) to induce the innate immune response [41]. Initial activation of TLRs triggers an inflammatory cytokine response, including the induction of *TNFα*, yet TTP is also induced by TLRs to harness the expression of these cytokines and control inflammation [10], [42]. However, subsequent activation of TLRs results in an impaired innate inflammatory response known as tolerance [38]. Thus, in *TTP*-low tumors with high levels of necrosis, it is likely that increased levels of DAMPs are present, which cause chronic stimulation of TLRs that leads to a tolerant state of the innate immune pathway and immune suppression. Further studies will determine if decreased levels of *TTP* cause the increased levels of necrosis, or if rather decreases in *TTP* levels reflect tolerance of the innate immune response.

Gene cluster analysis of kinetic profiles following LPS treatment of macrophages revealed that *TTP* and *ATF3* are coordinately and rapidly induced in response to TLR4-activated innate immunity [25]. Other genes similarly regulated include *DUSP1*, *EGR1*, *EGR2*, *JUN*, and *NR4A1* [25], which are components of the *TTP*-low tumor gene signature, again linking *TTP* levels and innate immunity. Similar to TTP function in controlling innate immunity, ATF3 transcriptionally represses the expression of cytokines, such as *IL-6* and *IL-12b*, to function as a negative feedback regulator of innate immune-driven inflammation [25]. This suggests that TTP and ATF3 together harness TLR-mediated inflammation, and future studies should include tests of their functional relationship and how this relates to tumor development and progression.

Other connections of CREB in *TTP-*low expressing tumors also bear further investigation. First, CREB and TTP may be linked via a common upstream regulator, for example p38 mitogen-activated protein kinase (MAPK). In LPS-treated macrophages, p38 activates its downstream kinase MK2, which stabilizes *TTP* mRNA and directly phosphorylates TTP protein regulating its subcellular localization and stability [13], [43]. Also p38 activates the mitogen- and stress-activated protein kinases MSK1 and MSK2 that, in turn, induce TTP protein [12]. In addition, MSK1 and MSK2 facilitate the stress-induced phosphorylation of CREB at Ser-133, which induces the transcription of several immediate early genes including *c-fos*, j*unB*, and *egr1* [44], which are, notably, part of the low-*TTP* tumor gene signature. Therefore, future studies should test if impairing p38 MAPK in tumor cells suppresses both *TTP* expression and CREB activity. In addition, given that LKB1 functions in part to prevent CREB target gene activation [45], the decreased mutation frequency of *STK11* (LKB1) in *TTP*-low lung adenocarcinomas compared to the *TTP*-high cohort corresponds with the observed decrease in CREB-target gene expression in *TTP*-low tumors. Furthermore, *TTP*-low tumors express a novel CREB family member, CREMΔZIP, that most likely functions as a dominant negative repressor of CREM by acting in a manner similar to ATF3ΔZIP, an ATF3 isoform that also lacks the leucine zipper domain. ATF3ΔZIP is unable to bind to DNA but functions as a dominant negative of ATF3 by competing for co-factors that ATF3 requires to repress transcription [40]. Collectively, these links between reduced *TTP* expression and repressed CREB activity in cancer support the idea that therapeutic CREB agonists, for example colforsin, salbutamol, clenbuterol, or isoprenaline, may show benefit as therapeutics for *TTP*-low expressing tumors, particularly ones where there is a decrease in CREB activity.

## Supporting Information

S1 Fig **Genes in the** ***TTP*** **-low tumor gene signature are also regulated by activation of innate immunity by LPS.** Gene expression profiling analysis of GSE32574 shows the expression levels of genes in the *TTP*-low tumor signature in unstimulated macrophages versus LPS-treated macrophages. All genes shown were expressed above the 50^th^ percentile in at least one sample.
(TIF)

S2 Fig **CREB family expression in lung squamous cell carcinoma and colon adenocarcinoma based on** ***TTP*** **levels.** Gene expression profiling showing the expression levels of canonical *CREB* family members in *TTP*-high and *TTP*-low expressing TCGA lung squamous cell carcinomas and colon adenocarcinomas.
(TIF)

S3 Fig **Expression of CREM splice variants in lung squamous cell carcinoma and colon adenocarcinoma based on** ***TTP*** **levels.** Gene expression profiling comparing the expression levels of *CREM* versus its dominant negative splice variants *ICER* and *CREM*Δ*ZIP* in *TTP*-high and *TTP*-low expressing TCGA lung squamous cell carcinomas and colon adenocarcinomas.
(TIF)

S1 Table **TCGA breast cancer tumors in the** ***TTP*** **-high and** ***TTP*** **-low quartiles.**
(XLSX)

S2 Table **TCGA lung adenocarcinomas in the** ***TTP*** **-high and** ***TTP*** **-low quartiles.**
(XLSX)

S3 Table **TCGA lung squamous cell carcinomas in the** ***TTP*** **-high and** ***TTP*** **-low quartiles.**
(XLSX)

S4 Table **TCGA colon adenocarcinomas in the** ***TTP*** **-high and** ***TTP*** **-low quartiles.**
(XLSX)

S5 Table **Differentially expressed genes in TCGA breast cancer dataset between high and low** ***TTP*** **-expressing tumors.**
(XLSX)

S6 Table **Differentially expressed genes in TCGA lung adenocarcinoma dataset between high and low** ***TTP*** **-expressing tumors.**
(XLSX)

S7 Table **Differentially expressed genes in TCGA lung squamous cell carcinoma dataset between high and low** ***TTP*** **-expressing tumors.**
(XLSX)

S8 Table **Differentially expressed genes in TCGA colon adenocarcinoma dataset between high and low** ***TTP*** **-expressing tumors.**
(XLSX)

S9 Table **Human cancer datasets with similarities to the** ***TTP*** **-low tumor gene signature.**
(XLSX)

S10 Table **Canonical pathways significantly altered by the** ***TTP*** **-low tumor gene signature.**
(XLSX)

S11 Table **Upstream regulators of the** ***TTP*** **-low tumor gene signature.**
(XLSX)
