# Supplementary figures and images for: CREB Targets Define the Gene Expression Signature of Malignancies Having Reduced Levels of the Tumor Suppressor Tristetraprolin
Source: PLoS One. 2014 Dec 26;9(12):e115517. doi: 10.1371/journal.pone.0115517 (PMC4277357; doi:10.1371/journal.pone.0115517)

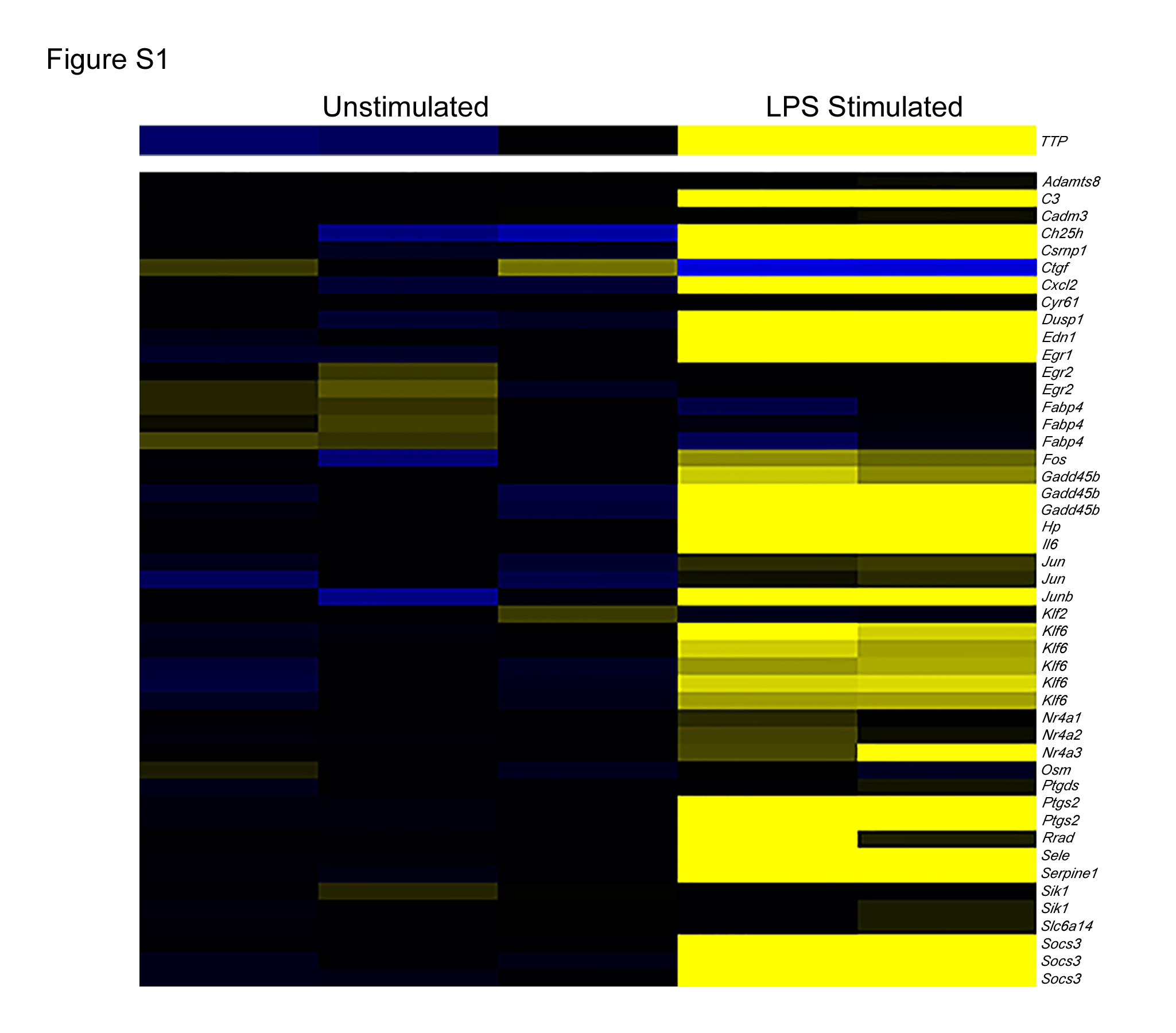

Supplement: S1 Fig — Genes in the TTP -low tumor gene signature are also regulated by activation of innate immunity by LPS. Gene expression profiling analysis of GSE32574 shows the expression levels of genes in the TTP-low tumor signature in unstimulated macrophages versus LPS-treated macrophages. All genes shown were expressed above the 50th percentile in at least one sample. (TIF) [file pone.0115517.s001.tif]

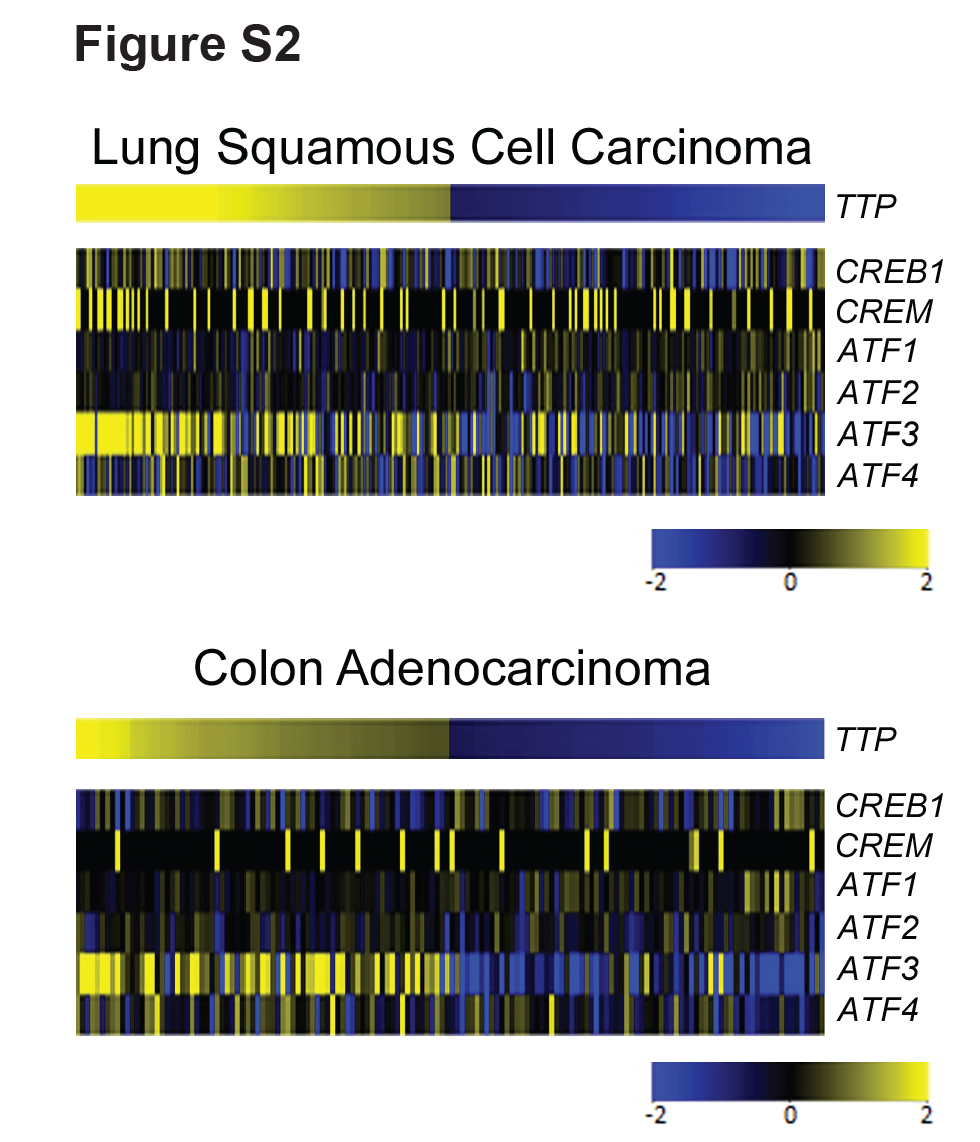

Supplement: S2 Fig — CREB family expression in lung squamous cell carcinoma and colon adenocarcinoma based on TTP levels. Gene expression profiling showing the expression levels of canonical CREB family members in TTP-high and TTP-low expressing TCGA lung squamous cell carcinomas and colon adenocarcinomas. (TIF) [file pone.0115517.s002.tif]

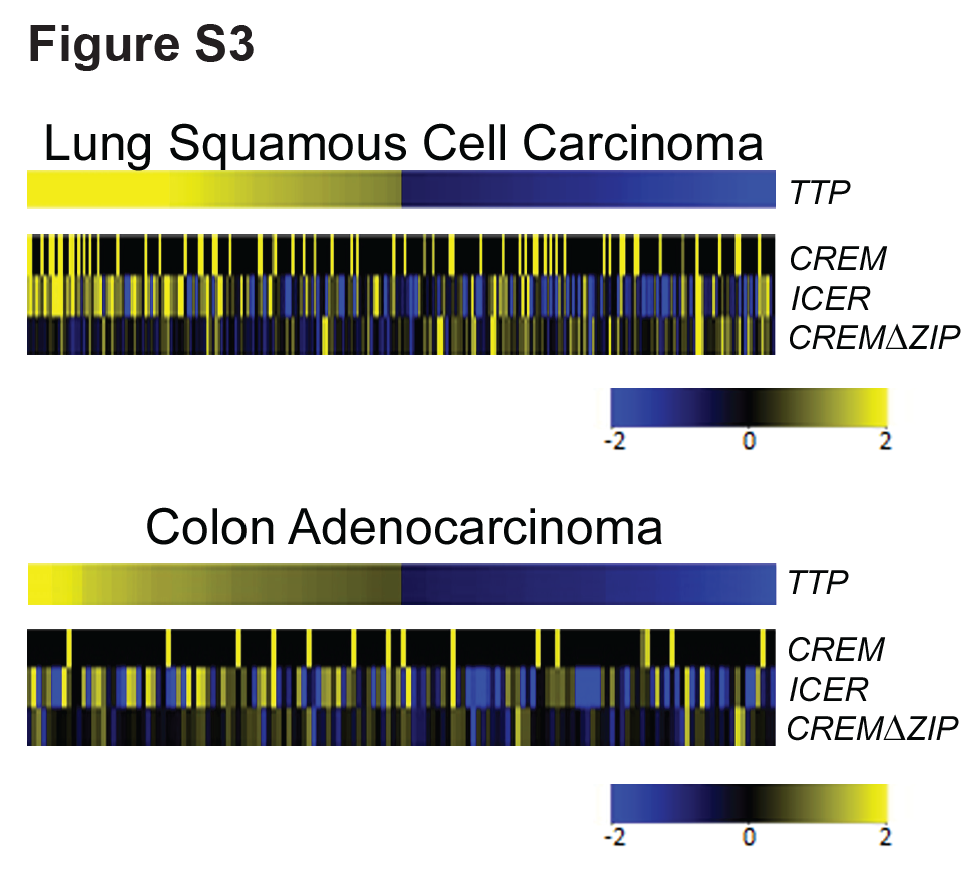

Supplement: S3 Fig — Expression of CREM splice variants in lung squamous cell carcinoma and colon adenocarcinoma based on TTP levels. Gene expression profiling comparing the expression levels of CREM versus its dominant negative splice variants ICER and CREMΔZIP in TTP-high and TTP-low expressing TCGA lung squamous cell carcinomas and colon adenocarcinomas. (TIF) [file pone.0115517.s003.tif]
